# Smooth Muscle Progenitor Cells: Friend or Foe in Vascular Disease?

**DOI:** 10.2174/157488809788167454

**Published:** 2009-05

**Authors:** Olivia van Oostrom, Joost O. Fledderus, Dominique de Kleijn, Gerard Pasterkamp, Marianne C. Verhaar

**Affiliations:** 1 Departments of Vascular Medicine, University Medical Center Utrecht, The Netherlands; 2 Experimental Cardiology, University Medical Center Utrecht, The Netherlands

**Keywords:** Smooth muscle progenitor cells, bone marrow, vascular disease.

## Abstract

The origin of vascular smooth muscle cells that accumulate in the neointima in vascular diseases such as transplant arteriosclerosis, atherosclerosis and restenosis remains subject to much debate. Smooth muscle cells are a highly heterogeneous cell population with different characteristics and markers, and distinct phenotypes in physiological and pathological conditions. Several studies have reported a role for bone marrow-derived progenitor cells in vascular maintenance and repair. Moreover, bone marrow-derived smooth muscle progenitor cells have been detected in human atherosclerotic tissue as well as in *in vivo* mouse models of vascular disease. However, it is not clear whether smooth muscle progenitor cells can be regarded as a ‘friend’ or ‘foe’ in neointima formation. In this review we will discuss the heterogeneity of smooth muscle cells, the role of smooth muscle progenitor cells in vascular disease, potential mechanisms that could regulate smooth muscle progenitor cell contribution and the implications this may have on designing novel therapeutic tools to prevent development and progression of vascular disease.

## INTRODUCTION

Vascular smooth muscle cells (SMC) are highly specialized cells whose principal function is contraction and regulation of blood vessel tone-diameter, blood pressure, and blood flow distribution [1]. However, SMC also play an important role in vessel wall pathology. The accumulation of SMC in the intima accompanies vascular diseases such as atherosclerosis, restenosis after angioplasty or stent placement and transplant arteriosclerosis and is therefore an important therapeutic target. In order to create novel therapeutic tools to prevent this, insight into the origin of SMC is essential. Both during embryonic development as well as in the development of vascular pathologies SMC originate from diverse sources. With regard to intimal SMC the longstanding theory is that changes in the environment lead to the migration of SMC from the media to the intima. However, other possible sources include adventitial fibroblasts or endothelial cells. Recent reports have indicated a role for bone marrow (BM)-derived smooth muscle progenitor cells (SPC) that can contribute to development and progression of vascular disease [2-5]. In this review we will discuss the heterogeneity of SMC, the role of SPC in neointima formation, possible mechanisms that could regulate SPC contribution and their potential as a target for new therapeutic strategies to prevent disease. 

## VASCULAR SMC IN HEALTH AND DISEASE

SMC are highly specialized cells that comprise the main component of the media of the vessel wall. Their principal physiological function is to maintain vasomotor tone via contraction or relaxation in response to a variety of metabolic and hormonal stimuli, as well as vessel integrity by proliferation and synthesis of extracellular matrix [6, 7]. Differentiated SMC are unique compared with other cell types as they display a low rate of proliferation, low synthetic activity, and express a unique repertoire of contractile proteins, ion channels, and signaling molecules required for the cell’s contractile function [7]. In contrast to either skeletal or cardiac myocytes that are terminally differentiated, SMC retain remarkable plasticity and can undergo rather profound and reversible changes in phenotype in response to changes in local environmental cues [7]. SMC accumulation in the neointima is a common feature in vascular diseases such as atherosclerosis, restenosis and transplant arteriosclerosis contributing to cardiovascular morbidity and mortality. 

Atherosclerosis is a chronic disease that progresses over years and comprises a complex interaction between lipids, endothelium, circulating and tissue inflammatory cells, platelets and SMC. Inflammatory mediators produced by activated macrophages can lead to phenotypic switching of medial SMC into a synthetic and migratory phenotype, which thereby form the fibrous component of the plaque. On the one hand, SMC contribute to plaque volume and stabilization by producing extracellular matrix, on the other hand they contribute to plaque degradation and rupture by producing growth factors and enzymes [8, 9].

Restenosis is a major problem after revascularization procedures such as angioplasty, stenting, and bypass grafting, characterized by intimal SMC recruitment, accumulation and inward remodeling. The acute mechanical injury triggers a cascade of events that includes endothelial denudation, direct SMC trauma, and the subsequent release of multiple growth factors that all play a role in the phenotypic switch of SMC. Interaction between inflammatory cells, platelets and SMC leads to the production of extracellular matrix by SMC which constitutes the bulk of the intimal lesion that contributes to restenosis [10-12]. 

Transplant arteriosclerosis can occur after heterotopic transplantation of organs or vessels. It is a major limitation of long-term survival of patients with solid organ transplantation. An immune-mediated response to donor endothelial cells and SMC is thought to take place. This leads to the infiltration of recipient mononuclear cells in the vessel wall of grafts at an early stage which secrete inflammatory cytokines inducing the recruitment and activation of SMC. Neointimal formation progressively occludes the vascular lumen, compromising luminal flow and leading to ischemic tissue damage [13, 14]. 

A diverse range of experimental animal models are used to study the role of SMC in vascular disease. The hyperlipidemia-induced mouse model develops spontaneous atherosclerosis which can be used in conjunction with a constrictive collar to accelerate and localize the development of lesions [15]. Restenosis is studied in animal models in which mechanical injury to the artery is induced by use of a wire, loose cuff or ligation [16]. Transplant arteriosclerosis involves heterotopic transplantation of organs or vessels. Although SMC recruitment and accumulation are a common feature of the above mentioned vascular pathologies, the mechanisms may differ according to the pathology and the cause and severity of injury. This is of importance when studying the characterization, origin and contribution of SMC to vascular disease in the different models. 

## HETEROGENEITY OF VASCULAR SMC

The contemporary paradigm is that dysfunctional endothelium and/or inflammatory cells produce growth factors, proteolytic agents, and extracellular matrix proteins that can induce migration of SMC from the media to the intima and promote the switch from a contractile to synthetic phenotype [8]. Studies on cytoskeletal and contractile protein expression in vascular development and disease have shown that expression of these genes is coordinately upregulated in differentiated SMC and downregulated in proliferating SMC [1]. However, this concept has been challenged by other studies showing that plaque SMC can be monoclonal [17, 18] or oligoclonal [19] suggesting that a predisposed SMC subpopulation is responsible for the production of intimal thickening. This indicates that SMC of the arterial wall could be biologically heterogeneous. Therefore, results may not be attributable to phenotypic variants of a single type of SMC, but reflect the activity of a mixture of different SMC subtypes. 

The description of contractile or synthetic phenotypes has contributed to the concept of SMC heterogeneity. In a mature blood vessel, medial SMC display a spindle-shaped contractile or differentiated phenotype characterized by the expression of contractile proteins specific to smooth muscle, such as smooth muscle α-actin (α-SMA), smooth muscle myosin heavy chain (SM MHC), caldesmon, calponin, SM22α and smoothelin which are important for the regulation of contraction in differentiated arteries [7, 12]. The most widely used SMC marker is α-SMA which is expressed at even early stages of development, and thus represents the most general marker of SMC lineage. Although α-SMA is permanently expressed in SMC, it is more abundant in contractile SMC than in synthetic SMC. Furthermore, myofilament bundles are abundant in the cytoplasm of the differentiated cell whereas organelles, such as rough endoplasmatic reticulum, Golgi and free ribosomes are few in number [6]. The synthetic SMC is typical of developing and pathologic arteries, and is characterized by an increased rate of proliferation, migration, and synthesis of extracellular matrix components. At the same time, synthetic SMC show a decrease in expression of smooth muscle-specific contractile markers [12]. Moreover, the cytoplasm contains large amounts of rough endoplasmatic reticulum, Golgi, and free ribosomes supporting its function in production of extracellular matrix [6]. Although intimal SMC share some similarities with medial SMC, studies have shown that they display several distinct characteristics regarding morphology, gene expression and synthetic properties [1, 20-22].

Another concept of relevance to SMC heterogeneity is that during embryonic development vascular SMC arise from multiple sources. This is of interest as SMC from different lineages could be functionally distinct. In the embryo, vessels and even different segments of the same vessel are composed of SMC populations that arise from distinct progenitors each with its own unique lineage. Studies have shown that there are eight origins from which SMC can be derived; neural crest, secondary heart field, somites, various stem cells, mesangioblasts, proepicardium, splanchnic mesoderm and mesothelium [23]. A better understanding of the origins of SMC in development and vascular disease could provide important new insights in neointimal SMC and therefore in new therapeutic strategies. 

## ORIGIN OF SMC IN VASCULAR DISEASE: ROLE OF BM-DERIVED SPC

**Fig. (1):**
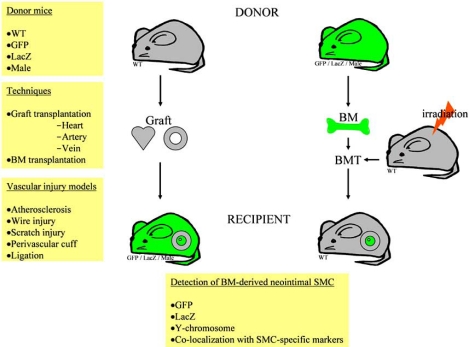
Contribution of BM cells to neointima after vascular injury or graft transplantation. BM cells in donor grafts or after vascular injury can be identified by immunohistochemistry, immunofluorescence or in situ hybridization for GFP, LacZ, or Y-chromosome and α-SMA. BMT, BM transplantation.

The traditional theory is that SMC migrate from the media into the intima in response to environmental cues. However, recent studies have shown that other origins for SMC exist including the adventitial layer of the vessel wall, the endothelium and the BM. In 1997, Asahara *et al. *[24] reported the existence and involvement of BM-derived endothelial progenitor cells (EPC) in adult physiological and pathological vasculogenesis. This opened up a new field of research exploring the possibility that BM cells, depending on environmental cues, can differentiate into different cell types. Whereas the involvement of BM-derived EPC in vessel repair and neovascularization has been acknowledged, the question whether BM is a source of SMC that contributes to neointima formation remains under debate (for an overview of studies see Table, Fig. **1**). 

### Variable Results of the Role of BM-Derived Cells in Vascular Disease

Several studies have suggested that circulating BM-derived cells contribute to neointima formation. In animal models of transplant arteriosclerosis including cardiac and aortic transplantation, SMC in the neointima have been shown to derive from host cells [2-4, 25-28]. Some of these studies, which involved BM-chimeric animals report that these cells are (in part) BM-derived [3, 4]. Heterotopic transplantation of wild-type hearts into BM-chimeric mice showed that after four weeks most of the neointimal cells in the coronary arteries were BM-derived [3]. Other studies, on the other hand, report no evidence for a contribution of BM to the recipient cells in the neointimal layer [25-27]. In mice models of vein and artery graft atherosclerosis, SMC in intimal lesions were reported to be derived from the recipients but not from BM-derived progenitor cells [26, 27]. 

In a hyperlipidemia-induced atherosclerotic mouse model, Sata *et al. *[3] showed that BM cells can be detected in atherosclerotic plaques. ApoE^-/-^ mice were transplanted with either GFP^+^ or LacZ^+^ BM cells and fed a high fat diet for eight weeks. A significant amount of α-SMA^+ ^cells in atherosclerotic plaques were GFP^+^ (40%) or LacZ^+^ (60%) respectively. Such BM contribution to atherosclerotic plaque composition could not be confirmed by Bentzon *et al*. [29]. In apoE^-/-^ mice that received BM from sex-mismatched GFP^+^ apoE^-/-^ mice and were fed a high fat diet they observed not one single GFP^+ ^α-SMA^+^ cell in atherosclerotic plaques of mice at 20 or 32 weeks of age. The same authors reported that in a model of plaque healing after spontaneous and mechanical plaque disruption in apoE^-/-^ mice, healing SMC were of local and not of blood origin [30]. 

Mechanical vascular injury leads to vascular remodeling involving accumulation of SMC at the site of injury. Studies have reported the contribution of BM cells to neointimal hyperplasia in this type of vascular disease [5, 31, 32]. In an elegant study by Tanaka *et al. *[5], the contribution of BM cells was investigated in a variety of mechanical vascular injury models. It was concluded that BM cells contribute to SMC accumulation caused by wire-mediated endovascular injury. Interestingly, Tanaka *et al. *[5] showed in two additional mechanical injury models that only a few BM cells were α-SMA^+^ in neointima after perivascular cuff placement and ligation of common carotid artery. Notably, BM cells were abundant in the neointima and media after carotid ligation, yet further characterization of these cells was not performed. Consistent with these results [5], it was reported that in a scratch injury model 56% of the neointimal cells was BM-derived of which 44% were SMC [31]. Moreover, Sahara *et al. *[32] showed the contribution of different BM cell populations after wire-mediated vascular injury. Total BM (TBM) cells, c-Kit^+ ^Sca-1^+ ^Lin^-^ (KSL) cells, or highly purified hematopoietic stem cells, expressing GFP, were transplanted into irradiated mice before injury. TBM and KSL cells could give rise to vascular cells and therefore contributed to neointimal and medial SMC after vascular injury. However, this did not occur in the group of mice that received a purified population of hematopoietic stem cells as hardly any GFP^+ ^cells were detected in the lesions. 

The variable results in studies investigating the role of BM in vascular disease suggest that the origin of intimal cells is diverse and that the mode or severity of injury can determine the contribution of BM-derived cells to neointimal SMC. Besides the differences in the pathogenesis of the employed experimental models, differences in methodology could explain in part the conflicting conclusions. Severity of injury may play a major role in the recruitment process of BM cells. Wire injury is known to induce complete endothelial denudation and medial cell loss as a result of apoptosis whereby the subsequent expression of growth factors may be important for the involvement of BM-derived cells [3, 33]. BM transplantation involving lethal irradiation followed by exogenous transplantation of BM cells could affect the involvement of BM cells in vascular remodeling. Indeed, it was recently described that BM transplantation had suppressive effects on neointima formation after wire injury [34]. Furthermore, although BM origin of neointimal SMC could not always be confirmed, BM cells may contribute to other cell populations in the neointima [3, 5, 26, 31]. Evidently, the role of BM-derived SPC is far from understood and future studies will shed more light on its involvement in vascular disease. 

The adventitial layer potentially harbors a population of stem cells that can also contribute to vascular remodeling. Hu *et al. *[35] demonstrated that abundant progenitor cells in the adventitia can differentiate into SMC that participate in lesion formation in vein grafts. This is supported by a study which recently reported that the adventitial layer possesses a niche of stem cell antigen (sca-1)^+^ SPC. Moreover, this population of progenitor cells differentiated into SMC-like cells *ex vivo *[36]. Progenitor cells have also been detected in the medial layer of the vessel wall. Progenitor cells isolated from the tunica media of mouse aortas by flow cytometry could acquire the phenotype of SMC in culture as measured by positivity for calponin, α-SMA and SM MHC [37]. The possibility of a stem cell niche in the vessel wall could be of importance in studies that report a non-BM-derived origin for cells in the neointima [5, 25-27, 29, 30, 38]. 

Human data on the origin of SMC in vascular disease is valuable yet scarce. Studies have described the detection of recipient cells in donor vessels after female to male organ transplantation. Grimm *et al. *[39] reported the presence of recipient cells in the vascular and interstitial compartments of renal allografts after female to male renal transplantation. Furthermore, Quaini *et al. *[40] showed a high number of recipient cells in eight hearts that were transplanted from females to males. Until now, one study has shown that sex-mismatched BM transplant subjects can have BM-derived SMC throughout the atherosclerotic vessel wall. Extensive recruitment of BM cells in diseased and not in undiseased segments was demonstrated and cell-cell fusion events were excluded as a cause for this enrichment [41]. In contrast, Yokote *et al.* [42] examined coronary artery autopsy specimens of two individuals who had undergone allogenic BM transplantation to determine the origin of vascular SMC in the vessel wall. None of the intimal SMC were of BM origin, yet in one individual donor-derived α-SMA^+ ^cells were detected in the media. The difference in underlying disease states of the patients may be the cause of the contradictory results. These studies suggest that BM progenitor cells may also play a role in human neointima formation. 

### Debatable Issues for BM-Derived Cell Characterization

A number of issues are controversial regarding the method by which BM cells are characterized. One of these issues regards the detection method of BM cells that express SMC markers in BM-chimeric animals. In these animals, BM cells are usually labeled with LacZ, GFP or are sex-mismatched. The specificity of co-localizing techniques such as low resolution immunomicroscopy to assess the origin of cells in BM-chimeric animals has been questioned. In view of this, a high-resolution confocal microscopy with Z-axis analysis has been employed to convincingly demonstrate that BM-derived cells express α-SMA in neointima after wire-mediated vascular injury [5]. On the other hand, Bentzon *et al* used this microscopy technique in a hyperlipidemia-induced atherosclerotic mouse model and showed no colocalisation of BM and SMC [29, 30]. High-resolution electron microscopy is another technique which has been used to confirm the presence of BM-derived SMC [3, 5]. Furthermore, to avoid the use of GFP antibodies which can potentially increase the risk of false signals by nonspecific antibody binding, studies have applied a plastic embedding technique of arteries to preserve endogenous GFP-fluorescence signal [5, 32]. 

Another debatable issue is the choice of markers to identify intimal SMC. α-SMA is widely used in most studies to identify SMC as it is considered a sensitive marker for SMC. However, α-SMA is not a definitive SMC lineage marker and has been reported to be expressed by many lineages other than SMC [1]. More specific markers including SM MHC, calponin, SM22, caldesmon and smoothelin may further confirm SMC presence. Nevertheless, whether intimal SMC, and more importantly, BM-derived neointimal SMC express these markers typical of “contractile” SMC is not clear. 

Much effort has been devoted to targeting migration and proliferation of medial SMC, yet to date no effective therapy exists. Investigation into the origin of SMC and the mechanisms that alter SMC phenotype will potentially shed light onto mechanisms that drive pathological arterial remodeling. Discrepancies still remain in interpretation of *in vitro* and *in vivo *data as different animal models, methodology and cell markers are used. Particularly, this must be kept in mind when extrapolating data to the human condition as pathologies differ. 

### Complex Role of Circulating SPC in Health and Disease

SMC can exhibit a wide range of phenotypes in response to changes in the local environment. Due to this plasticity, selecting unique markers that can identify SMC at different stages of differentiation, particularly in relation to vascular disease, is complex. This complexity is reflected in the determination and characterization of circulating progenitors for SMC and is experimentally challenging. 

In healthy conditions, SPC have been cultured from the mononuclear cell fraction of human peripheral blood in selection medium supplemented with platelet-derived growth factor (PDGF)-BB. Simper *et al. *[43] described for the first time the rapid outgrowth and expansion of SPC that were positive for α-SMA, SM MHC, and calponin. Furthermore, integrin α_5_β_1_ expression was increased and facilitated adhesion to extracellular matrix protein fibronectin. In a following study, the integrin profile of SPC in human peripheral blood was studied in more detail, as well as their adhesion to extracellular matrix *in vitro*. Moreover, injection of SPC into porcine coronary arteries showed that SPC can adhere *in vivo *to fibronectin-coated mesh stents [44]. Other culture conditions such as using collagen-coated plates or media supplemtented with growth factors such as transforming growth factor (TGF-β), fibroblast growth factor (FGF) and insulin-like growth factor (IGF) have also been used to identify a SPC population in the mononuclear cell fraction of peripheral blood [45, 46]. 

Circulating SPC have been identified in different disease states. In patients with type 1 diabetes, the number of collagen- and α-SMA- expressing SPC was increased after seven days of culture indicating a possible role in adverse tissue remodeling [47]. In peripheral blood of patients with coronary artery disease (CAD), circulating cells expressing CD14, CD105 and α-SMA were increased compared to non-CAD patients [45]. This shows a potential role for SPC in the pathogenesis of vascular disease. In addition, it was reported that in patients with atherosclerotic CAD, the number of peripheral CD34^+^ cells was increased one day after a stenting procedure which was independently predictive of instent restenosis [48]. Although the above mentioned studies identify circulating SPC both in healthy and disease conditions, they do not elucidate the potential biological relevance and actual contribution to vascular disease. Until now, one study has investigated the direct role of circulating SPC in atherosclerosis. Zoll *et al. *[49] reported that injection of human SPC, cultured from the mononuclear cell fraction of peripheral blood, into immune-deficient atherosclerotic-prone mice was able to limit plaque development and promote changes in plaque composition towards a stable phenotype. It is not clear from this study how SPC injection exerted these effects but possibilities include the incorporation of SPC themselves into the plaque or secretion of growth factors that stimulate local SMC and collagen synthesis. They also investigated SPC levels in patients with acute coronary syndrome compared to patients with stable angina. Blood-derived SPC were cultured for five weeks after which expression of SMC markers was analyzed. SPC levels in patients with acute coronary syndrome were reduced compared to stable angina patients suggesting that reduced SPC levels might play a role in plaque destabilization. Although the above-mentioned conflicting results may in part be due to differences in methods to identify circulating SPC, there is an indication for both beneficial as well as adverse roles for SPC. This may depend on the severity and cause of vascular injury, the presence of cardiovascular risk factors [47] and stage of atherosclerosis. At the early onset of atherosclerosis SMC contribute to intimal thickening, however, in advanced atherosclerosis, when a plaque consists of a large lipid core with an inflammatory component and a thin fibrous cap, SMC play a major role in the maintenance of plaque stability [9]. Therefore, SPC could be beneficial in atherogenesis as a factor promoting plaque stability and can thus be considered a ‘friend’ in vascular disease. In contrast, involvement of SPC in neointima formation highlights a detrimental role for SPC as ‘foe’ in vascular disease.

In peripheral blood of mice, a variety of markers have been used to identify SPC. One day after arterial injury, Zernecke *et al. *[50] found that the number of c-kit^-^/lin^-^/PDGF receptor (R)-β^+^/sca-1^+ ^cells was increased in peripheral blood. Furthermore, these cells were able to differentiate into SMC in response to PDGF-BB *in vitro* and respond to stromal cell-derived factor (SDF)-1α – mediated recruitment and differentiation into neointimal SMC *in vivo*. This was confirmed by another group in a carotid artery ligation model in which a peak in number of sca-1^+^/lin^-^/c-kit^-^ cells was seen in peripheral blood one week after injury and the number of sca-1^+ ^cells in the adventitia was increased at time points coinciding with neointima formation [51]. Other studies have confirmed the presence of a sca-1^+^ stem cell population in the adventitia which are able to differentiate into SMC in the presence of PDGF-BB [35, 36]. 

Besides peripheral blood, BM stromal cells are also able to differentiate into SMC *in vitro *[52]. A unique cell population was isolated from the adhesion fraction of BM cells based on expression of GFP that was driven by the SM22α promotor. These cells expressed PDGFR-β but neither mature nor immature SMC-specific proteins. By following the longitudinal transition of their phenotypes, it was found that they eventually differentiated into SMC as measured by expression of calponin, SM1 and α-SMA. Several studies indicate a role for PDGF in SPC differentiation and characterization [50]. The recruitment of BM cells that are able to differentiate into SMC, to perivascular sites in tumors [53] is dependent on PDGF-PDGFR-β signaling. Moreover, it is of importance in recruitment of SMC during blood vessel formation in the mouse [54]. 

Identification and characterization of SPC in tissue and in culture is a field of research that is just at the beginning of exploration. Many markers are not unique for different SMC phenotypes, which results in hurdles when applying this knowledge to the SPC field. Extensive characterization of SPC is required to evaluate their contribution to vascular remodeling and potential as a therapeutic target. 

## SPC AS A THERAPEUTIC TARGET IN VASCULAR DISEASE

SMC accumulation is a key event in neointima formation during atherosclerosis, restenosis and transplant arteriosclerosis. Inhibition of the contribution of SPC in neointima may therefore be a possible target for prevention of vascular disease. However, SMC accumulation in fibrous cap formation is desirable for plaque stability indicating a potential beneficial role for SPC. Although the exact role of SPC in vascular remodeling is not clear, a number of signaling pathways have been reported to be of importance in SPC recruitment and homing. This may be relevant when considering influencing the involvement of SPC in vascular disease. 

SDF-1α is a CXC chemokine which is essential in regulating hematopoietic progenitor cell mobilization [55, 56]. The importance of SDF-1α and its receptor CXCR4 has been shown in recruitment of progenitor cells in vascular remodeling. SDF-1α can play a role in neointima formation by regulating neointimal SMC content [57]. Moreover, the expansion of circulating PDGFR-β^+^/sca-1^+^/lin^-^ progenitor cells in the peripheral blood of apoE^-/-^ mice following arterial injury is mediated by SDF-1α. Regarding the receptor of SDF-1α, CXCR4 plays a pivotal role in this process as repopulation of apoE^-/- ^mice with CXCR4^-/-^ BM display a reduction in neointimal hyperplasia linked to a decrease in SMC content [50]. Interestingly, in a therapeutic setting, a CXCR4 antagonist can inhibit neointima formation and SPC mobilization after arterial injury [58]. Platelets may play an essential role in the SDF-1α/CXCR4 axis as they can secrete SDF-1α. Platelet adhesion to exposed subendothelium takes place after vascular injury and it has been shown that they can interact with progenitor cells [50, 59]. Inhibition of platelet adhesion, by inhibiting P-selectin, can abrogate adhesion of BM-derived progenitor cells at sites of endothelial disruption [59]. Therefore, platelet-dependent mechanism of SDF-1α-secretion could be essential in the involvement of SPC in arterial remodeling. 

Other factors have also been implicated in SDF-1α expression. Endothelial nitric oxide synthase (eNOS)-deficiency can increase SPC in association with upregulation of SDF-1α in arterial injury indicating an additional protective role of nitric oxide in vascular disease [51]. Furthermore, expression of the transcription factor hypoxia-inducible factor (HIF)-1α in injured arteries can control SDF-1α-mediated neointimal formation in apoE^-/-^ mice [60]. HIF-1α can induce SDF-1α expression resulting in an increase in adhesion, migration and homing of circulating CXCR4^+^ progenitor cells to ischemic tissue [61]. Moreover, depletion of phosphatase and tensin homology deleted on chromosome 10 (PTEN), a lipid phosphatase in SMC, results in HIF-1α-mediated production of SDF-1α in SMC. This can induce progenitor cell migration through a paracrine signaling mechanism. PTEN antagonizes phosphatidylinositol 3-kinase-mediated signaling events, resulting in an inhibitory effect on neointima formation in a carotid arterial balloon injury model [62]. Potentially, PTEN could thus be a target for inhibition of vascular remodeling [63].

In a clinical setting, current therapeutic strategies have been investigated regarding their role in SPC accumulation in neointima formation. Rosiglitazone, a peroxisome proliferator-activated receptor-γ (PPAR-γ) agonist, can inhibit intimal hyperplasia after balloon injury [64, 65]. This could be through promoting differentiation of progenitor cells towards the endothelial lineage and inhibiting differentiation towards the SMC lineage [66]. Similarly, sirolimus, which is used on stents to prevent in-stent restenosis, could attenuate the number of BM-derived SPC in lesions in a vascular injury model [67]. The same group reported that angiotensin II could accelerate, whereas the blocker of angiotensin II type 1 receptor suppressed neointima formation after arterial injury which correlated with the number of BM-derived SMC in the lesions [68]. 

**Fig. (2):**
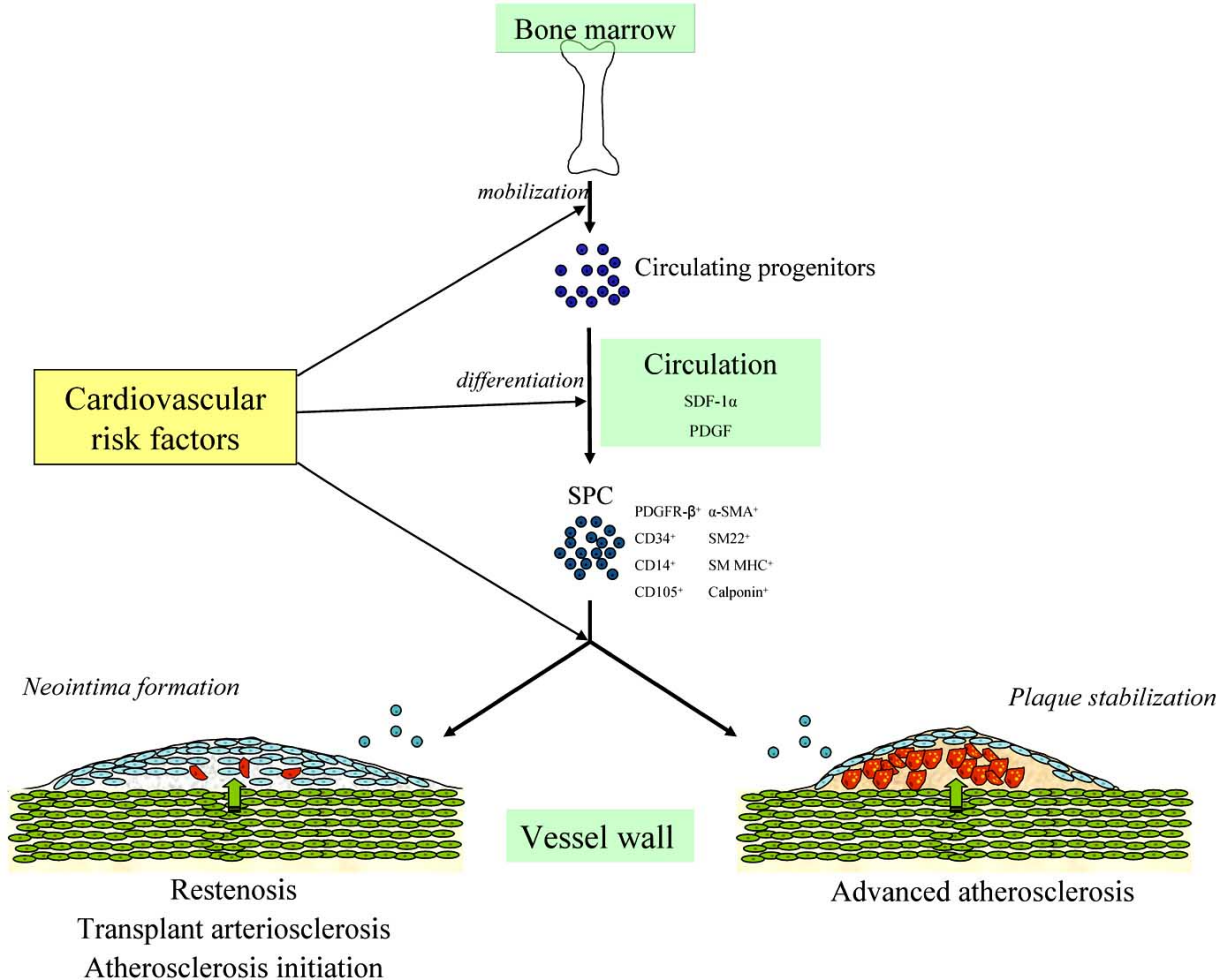
Potential dual role of SPC in vascular disease. Progenitor cells mobilized from the BM can differentiate, depending on environmental cues, into SPC that can play a role in vascular disease. On the one hand, SPC can contribute to neointima formation, on the other hand they can contribute to plaque stabilization which may be affected by the presence of cardiovascular risk factors.

Besides modulating differentiation of progenitor cells towards an endothelial phenotype, there may be a potential dual role for SPC in vascular disease which can be targeted. Until recently, it was thought that SPC have a detrimental role as a contributor to SMC accumulation in vascular disease. However, as described recently [49], SPC may be involved in plaque stability at advanced stages of atherosclerosis. Therefore one can imagine that targeting SPC to plaques in patients with advanced atherosclerosis can decrease the risk of plaque rupture. Indeed, it has been suggested that a deficiency in SPC could represent a novel risk factor in patients with CAD [49]. This indicates a role for SPC on the one hand as a ‘friend’ in an advanced stage of atherosclerosis and on the other hand as a ‘foe’ in restenosis after mechanical vascular injury and transplant arteriosclerosis (Fig. **2**). 

## CONCLUSION

Current animal and human data suggest that BM-derived SPC can give rise to SMC and participate in neointima formation. Signaling pathways involved in SPC differentiation, recruitment and homing are starting to be elucidated and cardiovascular pharmacological interventions can be developed to influence these processes. The exact role of SPC in the neointima is not clear and may differ depending on the cause and severity of vascular injury, stage of atherosclerotic disease and the presence of certain cardiovascular risk factors. These aspects may modulate mobilization, incorporation and differentiation of BM-derived progenitor cells, leading to SPC as ‘foe’ in vascular disease. However, SPC may potentially be a ‘friend’ as inducers of plaque stability in certain stages of atherosclerosis. Studies investigating BM involvement in vascular disease will contribute significantly to the knowledge of SMC accumulation that may result in novel therapeutic tools that can target and prevent development and progression of vascular disease.

## Figures and Tables

**Table 1:** Overview of Experimental Studies Investigating the Role of BM in Vascular Disease

Experimental Study	Technique	Method to Determine		Time	Results		Reference
		SMC Origin	SMC		Origin of Neointimal SMC	BM-Derived SMC	
*mouse*							
Transplant arteriosclerosis							
	Aortic transplantation	Y-chr/ISH	α-SMA/IH	30/60 days	Recipient	Few if any	Li *et al*. [25]
	Aortic transplantation	LacZ/X-gal staining	α-SMA/IH	8 weeks	Recipient	11% of intimal SMC	Shimizu *et al*. [4]
	Aortic transplantation	LacZ/X-gal staining	SM-LacZ transgenic/X-gal staining	2-6 weeks	Recipient	None	Hu *et al*. [26]
	Cardiac transplantation	LacZ/X-gal staining, GFP/IF staining	α-SMA/IF	4 weeks	Recipient (88%)	Majority of intimal SMC	Sata *et al*. [3]
	Vein graft transplantation	LacZ/X-gal staining	SM-LacZ transgenic/X-gal staining	8 weeks	Recipient (40%) and donor (60%)	None	Hu *et al*. [27]
Atherosclerosis							
	Hyperlipidemia-induced atherosclerosis	GFP/IF confocal, Y-chr/ISH	α-SMA/IH, Y-chr/ISH	Diet till 20 weeks/32 weeks of age	NA	None	Bentzon *et al*. [29]
	Spontaneous and mechanical plaque disruption	GFP/ IF confocal, Y-chr/ISH	α-SMA/IH, Y-chr/ISH	Up to 27 months of age/after 1 or 4 weeks	NA	None	Bentzon *et al*. [30]
	Hyperlipidemia-induced atherosclerosis	LacZ/X-gal staining, GFP/IF staining	α-SMA/IF/EM	8 week diet till 20 weeks of age	NA	58% or 42% LacZ^+ ^or GFP^+^ respectively of lesional SMC	Sata *et al*. [3]
Mechanical vascular injury							
	Wire-mediated endovascular injury	LacZ/X-gal staining, GFP/IF staining	α-SMA/IF, confocal	4 weeks	NA	26% of neointimal SMC, 35% of medial SMC	Tanaka *et al*. [5]
	Perivascular cuff	LacZ/X-gal staining, GFP/IF staining	α-SMA/IF, confocal	4 weeks	NA	A few neointimal SMC	Tanaka *et al*. [5]
	Ligation of carotid artery	LacZ/X-gal staining, GFP/IF staining	α-SMA/IH/IF, confocal	4 weeks	NA	A few neointimal SMC	Tanaka *et al*. [5]
	Scratch injury	Y-chr/ISH	α-SMA/IH	4 weeks	NA	44% of neointimal SMC	Han *et al*. [31]
	Arterial thrombus by insertion of suture into artery	Y-chr/ISH	α-SMA/IH	4 weeks	NA	None in arteries with minimal damage. Some BM-derived SMC in arteries with serious damage	Han *et al*. [31]
	Wire-mediated endovascular injury	GFP/IF staining	α-SMA/IF, confocal	4 weeks	NA	HSC did not contribute to neointimal SMC. BM and KSL cells did contribute partly to intimal and medial SMC	Sahara *et al*. [32]
*rat*							
	Stent implantation	R26-hPAP/IH, IF, confocal	α-SMA/IF, confocal	4 weeks	NA	None	Groenewegen *et al*. [38]
	Aortic transplantation	R26-hPAP/IH, IF, confocal	α-SMA/IF, confocal	2 months	NA	None	Groenewegen *et al*. [38]
*human*							
	Coronary artery atherosclerosis	Y-chr/ISH, blood type A, IH	α-SMA	10 years/90 days (n=2)	NA	In one patient possibly in media	Yokote *et al*. [42]
	Coronary artery atherosclerosis	Y or X-chr/ISH	α-SMA/IH	Between 41 and 1235 days (n=8)	NA	9.4% of intimal cells in females, 10.8% in males	Caplice *et al*. [41]

Y-chr, Y-chromosome; X-gal, X-galactosidase; hPAP, human placental alkaline phophatase; ISH, in situ hybridization; IH, immunohistochemistry; IF, immunofluorescence; EM, electron microscopy; HSC, hematopoietic stem cells; KSL, c-Kit^+^ Sca-1^+^ Lin^-^ cells; NA, not available
